# Phagocytic activity of peripheral blood and crevicular phagocytes in health and periodontal disease

**DOI:** 10.4103/0972-124X.65427

**Published:** 2010

**Authors:** K. Asif, Shaila V. Kothiwale

**Affiliations:** Department of Periodontics and Oral Implantology, K.L.E.S's Institute of Dental Sciences, Belgaum - 590 010, India

**Keywords:** Localized aggressive periodontitis, neutrophils, phagocytosis

## Abstract

**Background:**

Neutrophils constitute the main phagocytic cell system in mammalian host defense against an infecting agent. Abnormalities in leukocyte number and function are associated with increased susceptibility to periodontal diseases. The purpose of this study is to evaluate the *in vitro* phagocytic properties of crevicular and peripheral blood neutrophils in healthy and periodontitis subjects.

**Patients and Methods:**

A total of 30 subjects, that is, 10 patients in each of the following three groups: healthy controls, chronic periodontitis (CP), and localized aggressive periodontitis (LAP), were included in the study. The neutrophils were isolated from the peripheral blood and gingival crevice and tested for phagocytosis of *Candida albicans*. The percentage of leukocytes with ingested *C. albicans* was determined by light microscopy.

**Results:**

A significant reduction in the phagocytic activity of crevicular fluid polymorphonuclear neutrophils (CF-PMN) of LAP subjects (mean: 54.3±7)(*P*<0.001) was observed, compared to healthy controls (mean: 74.2±9) and chronic periodontitis subjects (mean: 69±9)(*P*=0.352). The mean percentage of peripheral blood polymorphonuclear neutrophils (PMNs) with phagocytosis of opsonized *C. albicans* in LAP patients was significantly reduced (mean: 74.9±5)(*P*<0.0068) compared to the phagocytic activity of neutrophils from controls (mean:82.1±3) and chronic periodontitis subjects (mean: 82.0±5)(*P*=0.970). There was no significant reduction in the phagocytic activity of CF PMNs (mean: 69±9) (*P*=0.35) and peripheral blood PMNs (mean: 82.5)(*P*=0.97) in the chronic periodontitis group when compared to the control group.

**Conclusion:**

The phagocytic activity of both crevicular and peripheral neutrophils in subjects with periodontitis is altered, increasing the susceptibility to periodontitis. Thus individual susceptibility may be an additional and important modifying factor in the pathogenesis of periodontal disease.

## INTRODUCTION

Periodontal diseases are infectious in origin. The extent and severity depends on the interaction between the pathogenic challenge and host response. A balance between both helps maintain periodontal homeostasis. Inadequate or excessive host response can lead to tissue destruction and such an outcome may be largely specific to the individual.

The presence of neutrophils in the gingival crevice serves to protect the periodontal tissue against microbial attack by phagocytosing the microorganisms or by releasing lysozomal enzymes into the crevicular environment extracellularly.[1] Patients with PMN defects, either quantitative or qualitative, are associated with increased susceptibility to rapid and often severe periodontal destruction.

Crevicular PMNs are functionally intact and in many respects comparable with peripheral blood PMNs. The concept of site-specificity of periodontal disease has led to the development of methods for obtaining gingival crevicular cells for study. One such technique, utilizing crevicular washing,[2] has been employed to assess the functional cellular and humoral immune components to be investigated.

Crevicular PMNs obtained from healthy sites have been found to be viable and capable of phagocytosis.[3] Most of the information concerning impaired neutrophil function and periodontal tissue destruction has been obtained from *in-vitro* studies, usually from peripheral blood PMNs.

### Aims

The aim of this study is to evaluate the *in-vitro* phagocytic properties of crevicular neutrophils and peripheral blood neutrophils in different forms of periodontitis; to correlate the altered neutrophil activity and the individual susceptibility to periodontitis.

## PATIENTS AND METHODS

This study was conducted in the Department of Periodontics, K.L.E.S's Institute of Dental Sciences, Belgaum, Karnataka. The subjects were explained about the study design and an informed consent was obtained. All the subjects were systemically healthy and none had received antibiotic therapy in the past six months. The periodontal examination included, full mouth periapical radiographs, gingival index (Loe and Silness 1963), plaque index (Silness and Loe 1964), and Russel's periodontal index (Russel A L 1956) [Table 1]. The subjects were divided into three groups, consisting of 10 subjects in each group, based on their periodontal status and according to the American Academy of Periodontology (AAP) classification of periodontal diseases, 1999.

**Table 1:** Mean (± SD) age, sex, and clinical parameters of control and disease groups

Group	M: F	Age	PI	GI	RPI
Control / healthy (A)	7:3	30:3 (± 7.25)	0.93 (± 0.0.42)	1.27 (± 0.52)	1.0 (± 0.49)
Chronic periodontitis (B)	5:5	42.9 (± 6.71)	2.11 (± 0.32)	2.22 (± 0.27)	3.35 (± 1.28)
Localized aggressive periodontitis (C)	5:5	17.1 (± 2.7)	1.12 (± 0.38)	1.35 (± 0.48)	2.15 (± 0.74)

Group A: Healthy controls with no evidence of bone loss or evidence of periodontitis, Group B: Chronic periodontitis patients, Group C: Localized aggressive periodontitis

In each case 2.0 ml of peripheral venous blood was drawn from the antecubital fossa with aseptic precautions, mixed with EDTA (Ethylene Diamine Tetra acetic acid) and transported to the laboratory. The red cells were allowed to sediment at room temperature. After a 30 to 60 minute period, the leukocyte rich plasma was isolated with the aid of a pipette and centrifuged at low speed (210 g) for 10 minutes. The neutrophils could be easily differentiated and were subjected to phagocytosis without any further treatment.[4]

Gingival crevicular cells were obtained from the crevice as follows. In each case 15 sequential washings of the gingival crevice were accomplished with phosphate buffered saline (PBS) using a 5 μl conventional pipette. The washes were collected in 1 ml Eppendorf-tubes and centrifuged at 2000 rpm for 10 minutes. The cells were washed twice with PBS and counted using a neubauer chamber.[5]

Suspensions containing peripheral blood, CF-PMNS, macrophages, and lymphocytes were adjusted to a concentration of 10^6^ cells/ml. *C. albicans* were used as indicator particles to determine the number of phagocytes containing and adhering *C. albicans*. The *C. albicans* were incubated at 26°C for 48 hours, washed with PBS and suspended in 6 ml PBS. The suspension was then heated in a water bath at 90°C for 30 minutes without destroying the morphological appearance of the *C. albicans*. They were subsequently washed twice with PBS, and suspended in PBS + 20% fetal calf serum (FCS) to a final concentration of 5 × 10^8^cells/ml.[5]

In each case a 0.2 ml suspension of heat-killed *C. albicans* was mixed with 0.2 ml of pooled human AB-serum for opsonization, for 30 minutes at 37°C, and suspended in 1 ml PBS + Fetal Calf Serum (FCS) (20%). Ten micro liters of this suspension (10^8^ /ml) was added to a 50 μl suspension (10^6^ /ml) of peripheral blood and crevicular cells. This suspension was mixed well on a microscopic slide and placed in a moist chamber for 30 minutes. Phagocytosis was stopped by draining the supernatant and covering the slide with 50 μl of stain solution, of 0.2 EosinY and 0.4% Trypan blue in PBS, and was examined with the help of a light microscope.[5]

**Figure 1:**
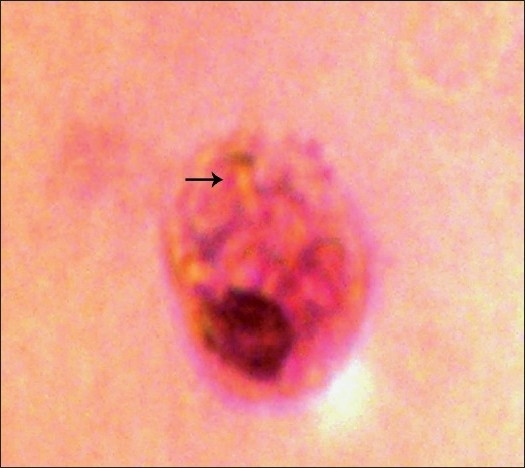
Phagocytosis of *C. albicans*

**Figure 2:**
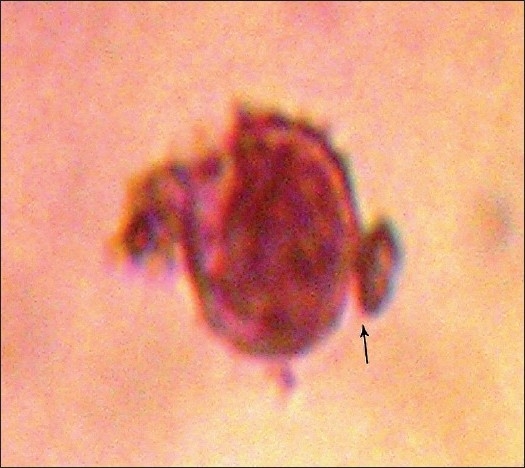
Adherence of *C. albicans*

The percentage of leukocytes with ingested *C. albicans* was calculated from the observations of 100 viable cells. [Figures 1 and 2].[5]

### Statistical analysis

Experimental results were expressed as mean ± standard deviation. The significance was evaluated using the Mann – Whitney Test (U test). The level of significance “*P*” value at 95% confidence interval was calibrated as: Nonsignificant (NS): *P*>0.05, Significant (S): 0.01 < *P*< 0.05 and highly significant (HS): *P*<0.001.

## RESULTS

The percentage of cells phagocytosing opsonized *C. albicans* and the number of phagocytosing *C. albicans* of LAP (mean: 54.3±7) were significantly lower (*P*<0.001) than the phagocytic activity of cells from the crevicular washings of the control group (mean: 74.2±9) [Table 2].

**Table 2:** Results of phagocytosis and statistical analysis (Mann Whitney Test)

Group	Crevicular PMN phagocytic activity		Peripheral blood PMN phagocytic activity	
	Mean ± standard deviation	*P* values	Mean ± standard deviation	*P* values
Control / healthy (A)	74.2±7.9		82.1±3.9	
Chronic periodontitis (B)	69.0±9.2	0.352†	82.0±5.3	0.9705†
Localized aggressive periodontitis (C)	54.3±7.98	0.001**	74.9±5.3	0.0068*

† Non significant

* Significant

** Highly Significant

When observed for peripheral blood PMN's activity, the percentage of peripheral blood PMN's with ingested *C. albicans* in LAP patients was significantly reduced (mean: 74.9±5, *P*=0.0068), when compared with the peripheral blood phagocytic activity of the control group (mean: 82.1±3) [Table 2].

There was no significant reduction in the phagocytic activity of CF PMNs (mean: 69.9±9, *P*=0.35) and peripheral blood PMNs (mean 82.5±5, *P*=0.97) in the chronic periodontitis group when compared with the control group.

## DISCUSSION

The present study examined the *in vitro* phagocytic properties of CF PMNs and PB PMNs in healthy and periodontitis subjects.

The results of phagocytosis of CF PMNs and PB PMNs were 74.2±7.9 and 82.17±3.9, respectively, in healthy controls [Table 2]. The CF PMNs activity was less compared to that of the peripheral blood PMNs. Wilton[3] and colleagues had reported similar mild reduction in the phagocytic function (72.2%) of CF PMNs recovered from healthy sites when compared with peripheral blood neutrophils.

In chronic periodontitis subjects there was no significant reduction in the phagocytic activity of CF PMNs and PB PMNs [Table 2] when compared to healthy controls. The result of the present study confirms previous reports about PMNs being functionally active in the gingival crevice of diseased sites.[5]

This study reported a highly significant decrease in the CF PMN activity in LAP patients as compared to controls [Table 2]. Neuman *et al*.[6] found no evidence of the ingestion of the test organism by the CF PMNs in any of the periodontosis subjects. Migratory capacity was lacking in CF PMNs, and transmission electro microscopy confirmed the degenerative state of most of these PMNs. Sigush *et al*.[5] reported a statistically significant reduction in the phagocytic capability of CF PMNs in subjects with LJP and rapidly progressive periodontitis when compared with controls and adult periodontitis. Murray and Patters[7] reported that the number and viability of the CF PMNs in the LJP lesion were similar to those in adult periodontitis, but their phagocytic activity was diminished.

Environmental factors in diseased pockets might result in locally diminished phagocytic activity of CF PMNs in LJP subjects. Direct interaction between the microorganisms and PMNs play a key role in the pathogenesis of periodontal disease.

Two of the organisms commonly associated with periodontitis are Porphyromonas gingivalis (*Pg*) and Aggregatibacter actinomycetemcomitans (*Aa*). *Aa* has been isolated from 75 – 100% of LJP lesions.[8]

One of the most studied virulence factor of *Aa* is leukotoxin. The toxin is a 116-Kda protein produced by 56% of the strains that have been isolated from LJP subjects.[9] It is a member of the RTX family of toxins that produce pore forming hemolysins or leukotoxins.[10] The toxin forms pores on the membranes of the neutrophils. The pores induced in the cells by leukotoxin, overwhelm the ability of the cells to sustain osmotic homeostasis, resulting in cell death. *Aa* also secretes a low molecular weight proteinaceous compound that inhibits PMN chemotaxis.[11]

Tolo *et al*.[12] demonstrated that molecules on the surface of *Aa* are associated with capsular material that is secreted into the medium, and binds to the Fc portion of IgG. This binding inhibits PMN opsonization and phagocytosis by 90%. Hence, the ability of this organism to secrete proteins that bind to the Fc receptor will inhibit phagocytosis.

The reason for the decreased activity of PMNs can also be attributed to the virulence of Pg. Along with the other bacteria it predominates the subgingival flora in LAP and refractory periodontitis subjects, at the diseased sites. Pg or enzymes derived from it have been shown to degrade TNF α and the macrophage colony stimulating factor, the one where PMN priming occurs.[13] Pg also impedes the migration of PMNs in response to formyl-methionyl-leucyl-phenylalanine (FMLP) and IL 8. PMNs incubated with supernatant products derived from Pg show decreased PMN phagocytosis and they alter the surface expression of Fc √R II and Fc √R III receptors.[14]

The peripheral blood PMN activity was also found to be reduced in LAP subjects. Cianciola *et al*.[15] reported a significant decrease in the PMN phagocytosis of opsonized S. aureus and chemotaxis in LJP patients. Cogen *et al*.[4] reported a significant decrease in the phagocytosis and chemotactic ability of peripheral blood PMNs in both LJP and generalized juvenile periodontitis (GJP) subjects. Suzuki *et al*,[16] stated that the radioisotope phagocytic indices were lower in a majority of the LJP subjects and the defect remained unchanged even after periodontal therapy. Neutrophil phagocytosis, chemotaxis, induction and spore germination were assessed in LJP patients. 79% of LJP and 58% of GJP subjects had chemotactic defects and 62% LJP and 29% GJP had phagocytic defects.[17]

Most of the antibodies against lipopolysaccharides and certain outer membrane proteins of *Aa* in individuals with LJP are of IgG2 subclass. Usually, IgG2 subclass antibodies against *Aa* are opsonic and promote phagocytosis of *Aa* by PMNs.[18]

Race may also predispose individuals to produce IgG2. IgG2 response is related to heightened disease susceptibility in patients with LJP. The reason IgG2 is related to disease susceptibility is due to pleomorphism in the Fc receptor affinity. A known allele of Fc√RII binds to IgG2 with relatively low affinity (R131) when compared with another allelic variant (H-131). PMNs from individuals who are homozygous for the H131 allele, normally phagocytose *Aa* in the presence of IgG2 derived from the sera of LJP subjects. Individuals with PMNs expressing low affinity receptor R131 are at risk, that is, the opsonic capability of IgG2 will be increased only at high concentration. Seventy three percent of the patients with LJP have been observed to be R131 homozygous. Thus, LJP may represent an area where there is a temporary inability of antibodies to effectively opsonize bacteria, resulting in disease.[19]

The following are the limitations of the present study: The decreased phagocytic activity by the cells *in vitro* might be due to the previous activity of these cells and a consequent decrease in the number of available cell surface receptors required for phagocytosis. Advanced ultra structural methods, including immunoelectron microscopy and radio labelled microorganisms that are detectable within the cell after phagocytosis, can be used in the study of the pathophysiology of CF PMNs.

## CONCLUSION

The present study shows that *in vitro* phagocytic activity of crevicular and peripheral blood neutrophils in LAP is reduced. Furthermore, it can be inferred that altered neutrophil function could severely weaken the host defense, resulting in the initiation and progression of periodontal disease.
